# sLZIP functions as a key modulator of bone remodeling by regulating the crosstalk between osteoblasts and osteoclasts

**DOI:** 10.1038/s12276-025-01414-3

**Published:** 2025-03-03

**Authors:** Sungyeon Park, Jeonghan Kim, Jesang Ko

**Affiliations:** 1 Division of Life Sciences, Korea University, Seoul, South Korea; 2 Department of Biochemistry, College of Medicine, The Catholic University of Korea, Seoul, South Korea; 3 Department of Medical Sciences, Graduate School of The Catholic University of Korea, Seoul, South Korea

**Keywords:** Osteoporosis, Gene therapy

## Abstract

Human small leucine zipper protein (sLZIP) regulates the differentiation of both osteoblasts (OBs) and osteoclasts (OCs). However, the regulatory role of sLZIP in bone remodeling and its involvement in bone disorders remain unclear. Here we investigated the role of sLZIP in bone remodeling and its importance in the development of cell therapies for bone diseases. sLZIP increased bone mass in an osteoporosis mouse model. Moreover, bone mass was lower in mesenchymal stem cell-specific murine LZIP-1/2 knockout (Osx-LZIP-1/2^fl/fl^) mice than in control LZIP-1/2^fl/fl^ mice. Compared with control mice, Osx-LZIP-1/2^fl/fl^ mice presented delayed bone fracture healing in osteoporosis. Conditioned medium from OBs differentiated from adipose-derived stem cells from Osx-LZIP-1/2^fl/fl^ mice attenuated OC formation and the migration of bone marrow-derived macrophages. However, conditioned medium from OCs from sLZIP transgenic mice induced OB differentiation and migration. sLZIP regulates the secretion of OC-derived sphingosine-1-phosphate, which induces OB differentiation. sLZIP also regulates OB-derived WNT16, which inhibits OC differentiation. Therefore, sLZIP is a key modulator of the crosstalk between OBs and OCs and promotes bone remodeling and fracture healing in osteoporosis. In addition, sLZIP-overexpressing adipose-derived stem cells promote bone formation and repair in osteoporosis. sLZIP is an excellent target for stem cell-based treatment of osteoporosis.

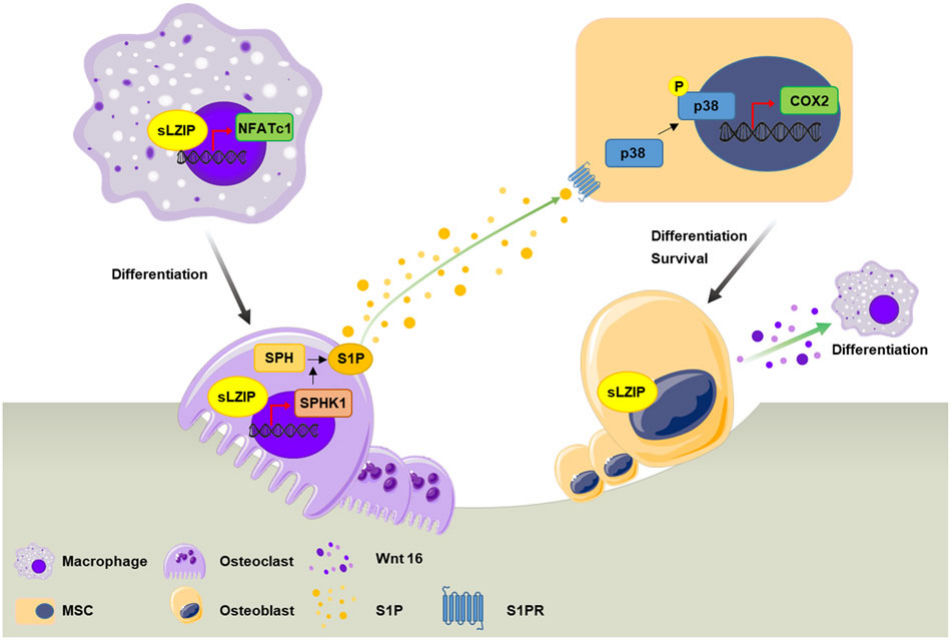

## sLZIP enhances bone remodeling and fracture healing

Bone remodeling is a natural process where old bone is replaced with new bone. This process can become imbalanced, leading to diseases such as osteoporosis. This study explores the role of sLZIP in bone health. Researchers used mice to study how sLZIP affects bone formation and healing. They found that sLZIP helps in forming new bone and aids in healing fractures. The study involved creating bone defects in mice and observing the effects of sLZIP on bone repair. Using various laboratory techniques to measure bone density and structure, the authors show that sLZIP promotes bone growth and helps repair fractures by enhancing communication between cells that build bone and those that break it down. The researchers conclude that sLZIP could be a promising target for developing new treatments for osteoporosis.

This summary was initially drafted using artificial intelligence, then revised and fact-checked by the author.

## Introduction

Bone remodeling is a continuous growth process that maintains bone structure by removing and repairing damaged bone and regulating mineral homeostasis through bone-resorbing osteoclasts (OCs) and bone-forming osteoblasts (OBs)^1^. An imbalance in bone remodeling leads to aging-related bone diseases such as osteoporosis, followed by bone porosity and fragility fractures^2^. Osteoporosis treatments are classified into two categories: anabolic treatments, which generate fresh bone tissue, and antiresorptive treatments, which reduce bone resorption and inhibit bone turnover to stimulate OBs^3^. Parathyroid hormones are used as anabolic treatments for osteoporosis; however, their use is limited to 2 years due to adverse effects^4^. Bisphosphonates were as the first antiresorptive agents used for osteoporosis; however, their long-term use causes osteonecrosis in the jaw and increases the risk of esophageal cancer^5,6^. Denosumab, a specific receptor activator of nuclear factor-κB ligand (RANKL) monoclonal antibody, inhibits bone resorption, but patients treated with denosumab often suffer side effects such as dyspnea, back pain, muscle pain and bone pain^7^. In this context, dual-function drugs targeting both mechanisms might have superior therapeutic effects; however, no such medication is currently available.

Bone remodeling involves the constant regeneration and resorption of bones to maintain bone homeostasis. Bone remodeling consists of three phases: (1) the initiation of bone resorption by OCs in the damaged region of the bone, (2) the transition from catabolism to anabolism and (3) bone formation by OBs. The conversion phase of the remodeling cycle refers to the process linking bone resorption and formation, termed coupling^8^. There are two types of coupling factors: those derived from OCs and those derived from OBs. These coupling factors serve as messengers between OBs and OCs during bone remodeling^9^. The OC-derived coupling factors sphingosine-1-phosphate (S1P), WNT10 and collagen triple helix repeat containing 1 regulate bone formation through crosstalk with OBs^10^. S1P is synthesized via phosphorylation by sphingosine kinase 1/2 (SPHK1/2) and plays an important role in bone development and metabolism^11,12^. OB-derived coupling factors, including RANKL, osteoprotegerin, semaphorin-3A, sclerostin and WNT16, communicate with OCs and induce bone resorption^13–15^. Therefore, a better understanding of the molecular mechanisms regulating bone remodeling and the crosstalk between OBs and OCs is important for developing better approaches for preventing and treating metabolic bone disorders.

The human small leucine zipper protein (sLZIP) is an isoform of LZIP (also known as CREB3). The murine isoforms of sLZIP and LZIP are LZIP-1 and LZIP-2^16^. sLZIP functions as a transcription factor that controls the proliferation, migration and invasion of various cancer cells^17–19^. sLZIP also regulates the differentiation of both OBs and OCs^20,21^. sLZIP induces OB differentiation by serving as a coactivator of runt-related transcription factor 2 (RUNX2), a key factor in OB differentiation and a corepressor of peroxisome proliferator-activated receptor γ2 (PPARγ2), which regulates adipocyte and OB differentiation^20^. sLZIP also increases the transcriptional activity of nuclear factor of activated T cells, cytoplasmic 1 (NFATc1) in bone marrow-derived macrophages (BMMs), leading to OC differentiation^21^. Although sLZIP plays an important role in both OB and OC differentiation, the molecular mechanism of bone remodeling remains unknown. In this study, we investigated the regulatory role of sLZIP in bone remodeling and its efficacy in the development of therapeutic agents for metabolic bone disorders.

## Materials and methods

### Cell culture and differentiation

Mouse adipose-derived stem (ADS) cells were isolated via sequential collagenase digestion of fat pads obtained from 8-week-old mice and grown in Dulbecco’s modified Eagle medium (DMEM) supplemented with 10% fetal bovine serum (FBS), 100 U/ml penicillin and 100 μg/ml streptomycin. When the cells reached confluence, OB differentiation was initiated with DMEM containing 50 μg/ml ascorbic acid and 10 mM β-glycerophosphate for 7–14 days. The differentiation medium was replaced every 2–3 days. Primary BMMs were isolated from the long bone-derived bone marrow of mice and maintained in α-MEM supplemented with 10% FBS, 100 U/ml penicillin and 100 μg/ml streptomycin. After 24 h, floating mouse primary bone marrow cells were separated into two 100 mm culture dishes and treated with 50 ng/ml macrophage colony-stimulating factor (M-CSF) for 3 days. To generate OCs, BMMs were seeded into 12- or 96-well plates containing 50 ng/ml M-CSF for 24 h and then treated with 30 ng/ml M-CSF and 50 ng/ml RANKL. The medium and cytokines were replaced every 2 days.

### RNA isolation and qRT‒PCR

Mouse primary BMMs were seeded into 12-well plates at a density of 1.5 × 10^5^ cells/well. After RANKL treatment, the cells were rinsed with PBS and detached. Mouse ADS cells were seeded into 12-well plates at a density of 8 × 10^5^ cells/well. After reaching 80% confluence, the cells differentiated into OBs for 7 days. Total RNA was isolated via a TaKaRa miniBEST universal RNA extraction kit (Takara Bio, Inc.) and cDNA was synthesized from total RNA using PrimeScript RT master mix (Takara Bio, Inc.) according to the manufacturer’s instructions. Quantitative PCR with reverse transcription (qRT–PCR) was performed on a LightCycler 480 II (Roche) using Evagreen-Express master mix (Applied Biological Materials). Semi-quantitative RT–PCR was performed on a T100 thermal cycler (Bio-Rad) using Pfu Plus 5× PCR Master Mix (Elpis Biotech, Inc.). The primer sequences used for PCR are listed in Supplementary Table 1.

### ARS and ALP staining and activity assay

Mouse ADS cells were differentiated in osteogenic medium for 14 days. Differentiated OBs were fixed with 4% formaldehyde solution for 10 min at 25 °C and stained with alizarin red S (ARS) and alkaline phosphatase (ALP) staining solutions. For ARS staining, the cells were washed with PBS, and cell matrix mineralization was evaluated by staining with 2% ARS solution (Millipore Sigma). For ALP staining, the cells were washed with PBS and stained using a TRACP/ALP double-staining kit (Takara Bio, Inc.) according to the manufacturer’s instructions. For ALP activity analysis, cells were seeded at a density of 4 × 10^4^ cells/well into a 96-well culture plate and cultured in osteogenic medium in a time-dependent manner. The cells were evaluated using a TRACP/ALP assay kit (Takara Bio, Inc.).

### TRAP activity and bone resorption assays

For the tartrate-resistant acid phosphatase (TRAP) activity assay, BMMs were seeded into 96-well culture plates at a density of 1 × 10^4^ cells/well. After 3 days of OB differentiation, the cells were washed with PBS and evaluated using a TRACP/ALP assay kit (Takara Bio, Inc.) according to the manufacturer’s instructions. For the bone resorption assay, the same number of cells were seeded on Corning osteo assay surface 96-well plates (Corning). Five days after OC differentiation, the cells were detached with 10% bleach solution for 5 min at 25 °C. After washing several times with double distilled water (DDW), the plates were placed in an incubator for 24 h. To quantify resorption activity, the resorption area (pixels)/total well area (pixels) for five random fields was determined.

### CM collection

To collect conditioned medium (CM), mouse BMMs were seeded in 12-well plates at a density of 1.25 × 10^5^ cells/well. The cells were differentiated into OCs for 5–7 days using RANKL (50 ng/ml) and M-CSF (30 ng/ml). After OC differentiation, the supernatants were collected and centrifuged at 200*g* for 3 min. To obtain OB-CM, mouse ADS cells were seeded in 12-well culture plates at a density of 8 × 10^4^ cells/well and differentiated into OBs for 7 days. The supernatant was subsequently centrifuged at 300*g* for 3 min.

### Transwell cell migration

Mouse ADS cells (2 × 10^4^ cells/100 μl) were seeded in the top chamber of 24-well plates (8 μm pore size; BD Biosciences). After 5 h, OC-CM was added to the bottom chamber for 18 h. Mouse BMMs (8 × 10^4^ cells/100 μl) were seeded into the top chamber of transwell plates with M-CSF (30 ng/ml). OB-CM was added to the bottom chamber for 24 h. Cells on the lower surface of the membrane were stained with 0.05% crystal violet according to the manufacturer’s protocol. The adherent cell counts were obtained from five randomly selected fields per well, and the values are expressed as the average of the counts.

### Cell proliferation

The cell proliferation assay was performed using EZ-cytox (DoGenBio), according to the manufacturer’s protocol. Mouse ADS cells were seeded into 96-well plates at a density of 3 × 10^3^ cells/100 μl. After 24 h, the cells were incubated with OC-CM for the indicated times. After the medium was removed, the cells were treated with 10 μl of EZ-Cytox solution and incubated at 37 °C for 30 min. Cell viability was determined by measuring the absorbance at 450 nm.

### Sphingosine kinase 1 activity assay

Sphingosine kinase 1 activity was determined using a sphingosine kinase activity assay kit (Echelon), according to the manufacturer’s instructions. Mouse BMMs were seeded into 12-well culture plates at a density of 1.5 × 10^4^ cells/well. After 24 h, the BMMs were differentiated into OCs in α-MEM containing 30 ng/ml M-CSF and 50 ng/ml RANKL for 7 days. The medium and cytokines were replaced every 2 days. Protein extracts (30 μg) were incubated with reaction buffer, 100 μM sphingosine and 10 μM ATP for 30 min at 37 °C. Sphingosine kinase 1 activity was measured using a SpectraMax i3x (Molecular Devices).

### Coculture of OBs and OCs

Mouse BMMs and ADS cells were cocultured indirectly or directly. To examine the crosstalk between OBs and OCs, indirect coculture was performed using either OB-CM or OC-CM. The CM of each cell line was transferred to wild-type (WT) ADS cells or BMMs for 10–13 days. Indirect coculture models were evaluated using ALP and TRAP staining. For direct coculture, ADS cells were cocultured with BMMs in 96-well plates in DMEM containing M-CSF (30 ng/ml) at an OB:OC ratio of 1:1.5. The cocultures were incubated in either OB or OC differentiation medium until 80% confluence was reached. After 7–10 days of coculture, the cells were evaluated using ALP or TRAP staining.

### S1P ELISA

BMMs were isolated from WT and sLZIP transgenic (TG) mice and differentiated for 7 days. OC-CM was collected, and S1P levels were measured using a mouse S1P enzyme-linked immunosorbent assay (ELISA) kit (MyBioSource, Inc.), according to the manufacturer’s instructions.

### Generation and transduction of sLZIP-overexpressing lentivirus

The sLZIP-overexpressing lentivirus was obtained from Sirion Biotech. The codon-optimized sequences of mouse CXCR4 and human sLZIP were cloned and inserted into the pcLV-CMV-eGFP-T2A-Puro-WPRE plasmid. Lentiviral vector (LV)-containing supernatants were produced by transient transfection of HEK293T cells. The titer was 10^9^ TU/ml. For transduction, ADS cells were seeded into 100 mm culture plates at a density of 1 × 10^6^ cells/plate. After 24 h, the cells were incubated in 5% FBS DMEM supplemented with 8 μg/ml polybrene and LV-CXCR4/sLZIP. The medium was changed after 16–24 h, and infected ADS cells were selected following continuous incubation in DMEM supplemented with 10 μg/ml puromycin for 24 h. Before the experiments, the mesenchymal stem (MS) cells were cultured for 48 h without puromycin.

### OVX surgery mouse model

Eight-week-old female mice were randomly divided into two groups: the sham control group (*n* = 4–5) and the ovariectomy (OVX) surgery group (*n* = 6–7). The female mice were ovariectomized under inhalation anesthesia induced using 1.8% isoflurane at a rate of 50 ml/min with 1–2% vaporization in the nose. After the hair in the dorsal mid-lumbar area was shaved, the dorsal skin was incised and the bilateral ovaries were removed. The wounds were closed with sutures. Sham control mice underwent the same procedure, but the ovaries were not removed. After 6 weeks, the OVX mice were euthanized for further analysis.

### Bone defect mouse model

A bone defect model was generated in 6–8-week-old mice. Before surgery, anesthesia was induced using a Somnosuite low-flow anesthesia system (Kent Scientific Co.) and isoflurane (Hana Pharm Co.). Isoflurane was used at 150–200 ml/min with 2.5% vaporization in the induction chamber. Anesthetized mice were maintained with 50 ml/min isoflurane and 1–2% vaporization via the nose. The right thigh was epilated and sterilized with 70% ethanol and the skin was incised over the lateral femoral aspect to expose the muscle. Blunt dissection of the quadriceps was performed to expose the femoral bone. The posterior and anterior cortices of the middle of the femur were perforated (0.6 mm in diameter) by using a drill bit and hand drill (Jeungdo Bio&Plant Co., Ltd.), while the site was irrigated with chilled PBS to prevent heat damage. After perforation, the muscle and skin tissues were repositioned using sutures. The surgical field was disinfected with povidone. The mice were then returned to their cages and administered an analgesic (30 mg/ml ibuprofen diluted in drinking water).

### Intravenous injection of ADS cells

For ADS cell-based therapeutic experiments, ADS cells from WT and TG were collected at 1 × 10^6^ cells/100 μl of PBS immediately before intravenous injection. For the LV-CXCR4/sLZIP-ADS cell-based therapeutic experiments, ADS cells from WT and ADS cells infected with LV-CXCR4 or LV-CXCR4/sLZIP were collected at 1 × 10^6^ cells/100 μl of PBS immediately before intravenous injection.

### μCT scanning

Mouse femurs were collected after anesthesia. Whole muscles and connective tissues were removed, and the samples were fixed in a 4% paraformaldehyde solution for 1 week at 4 °C. The femurs were subjected to micro-computed tomography (μCT) scanning using SkyScan1172 (Bruker) under the following conditions: 70 kV voltage, 135 μA current, 0.5 mm Al filter and 680 ms exposure time. The images were processed using reconstruction software (NRecon v1.6, Data Viewer v1.5) and three-dimensional (3D) visualization software (CTvox v2.6), and data analysis was performed using analysis software (CTAn v1.14). After 3D reconstruction, the trabecular bone volume/total bone volume (BV/TV) of a 1.604 mm length of cortical bone containing the defect region was automatically calculated using built-in software.

### Histological analysis

Mouse femurs were fixed in 4% paraformaldehyde (Sigma-Aldrich) solution at 4 °C. After fixation, the samples were decalcified at 4 °C for 2 weeks using 5 ml of OsteoSoft solution (Sigma-Aldrich). However, the samples used for OB staining were not decalcified. The solution was changed every 2–3 days. The samples were soaked in a 30% sucrose (Sigma-Aldrich) solution at 4 °C for 24 h and then transferred to the optimal cutting temperature mixture (Tissue-Tek, Sakura Finetek). The samples were then cryosectioned into 10-μm-thick slices proximal to the distal vertical direction and transferred to glass slides. The optimal cutting temperature compound was removed using ethanol and the samples were subjected to TRAP and ALP staining. After mounting in medium and applying cover slips, the samples were observed and analyzed under a light microscope.

### Statistical analysis

All the data are presented as the means ± s.e.m. Statistical analyses were performed using GraphPad Prism 5 (GraphPad Software). Two-tailed Student’s *t*-test results with *P* < 0.05 were considered statistically significant.

## Results

### sLZIP promotes bone formation and inhibits bone mass reduction in osteoporosis

**Fig. 1:**
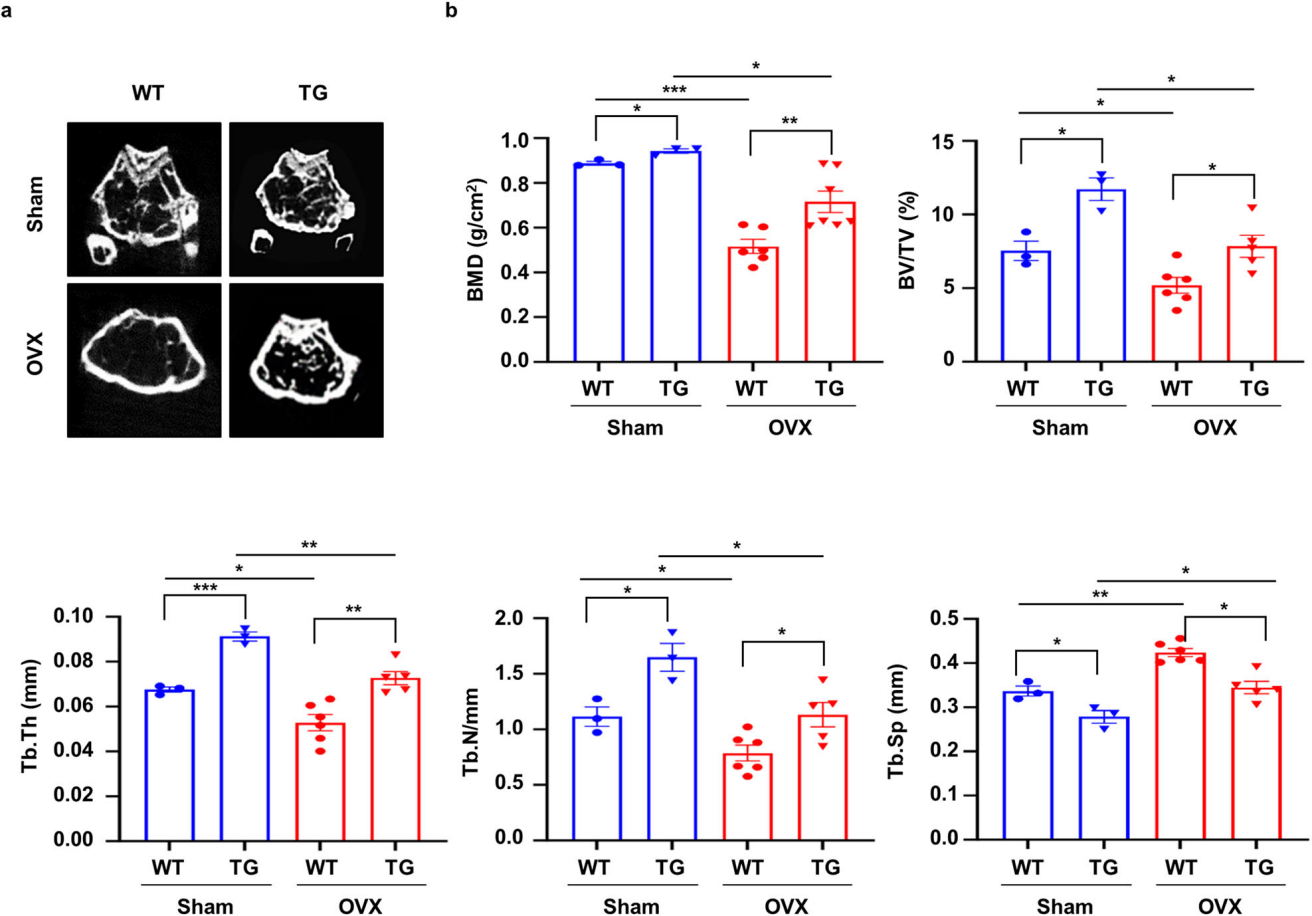
sLZIP promotes bone formation and inhibits bone mass reduction in osteoporosis. **a**, μCT of trabecular bone in the femurs of WT (*n* = 3) and sLZIP TG (*n* = 6) mice was performed. Representative μCT 2D images are shown. **b**, BMD, BV/TV, Tb.Th, Tb.N and Tb.Sp were quantified via μCT image analysis. Error bars show the mean ± s.e.m. *P < 0.05, **P < 0.01 and ***P < 0.001 (unpaired, two-tailed Student’s *t*-test).

sLZIP induces OB differentiation by inhibiting PPARγ2, a negative regulator of OB differentiation, and promotes bone formation^20^. Therefore, we investigated the effects of sLZIP on bone formation in a mouse model of osteoporosis. To investigate whether sLZIP is involved in the regulation of bone mass in osteoporosis, we generated osteoporosis model mice via OVX. Compared with sham mice, OVX mice presented a uterine atrophy phenotype and uterine weight reduction (Supplementary Fig. 1a). The results of μCT analysis revealed osteoporosis induced by OVX surgery, characterized by lower bone mass in OVX mice than in sham mice (Fig. 1a). Compared with WT mice, sLZIP TG mice presented greater bone mineral density (BMD), BV/TV, trabecular thickness (Tb.Th) and trabecular bone number (Tb.N), whereas trabecular separation (Tb.Sp) was lower in sLZIP TG mice than in WT mice (Fig. 1a, b). The bone mass of the OVX sLZIP TG mice was restored to the level observed in the sham WT mice (Fig. 1a, b). These results indicate that sLZIP promotes bone formation and inhibits bone mass reduction in osteoporosis.

### Deletion of sLZIP reduces bone mass and impedes osteoporosis recovery

**Fig. 2:**
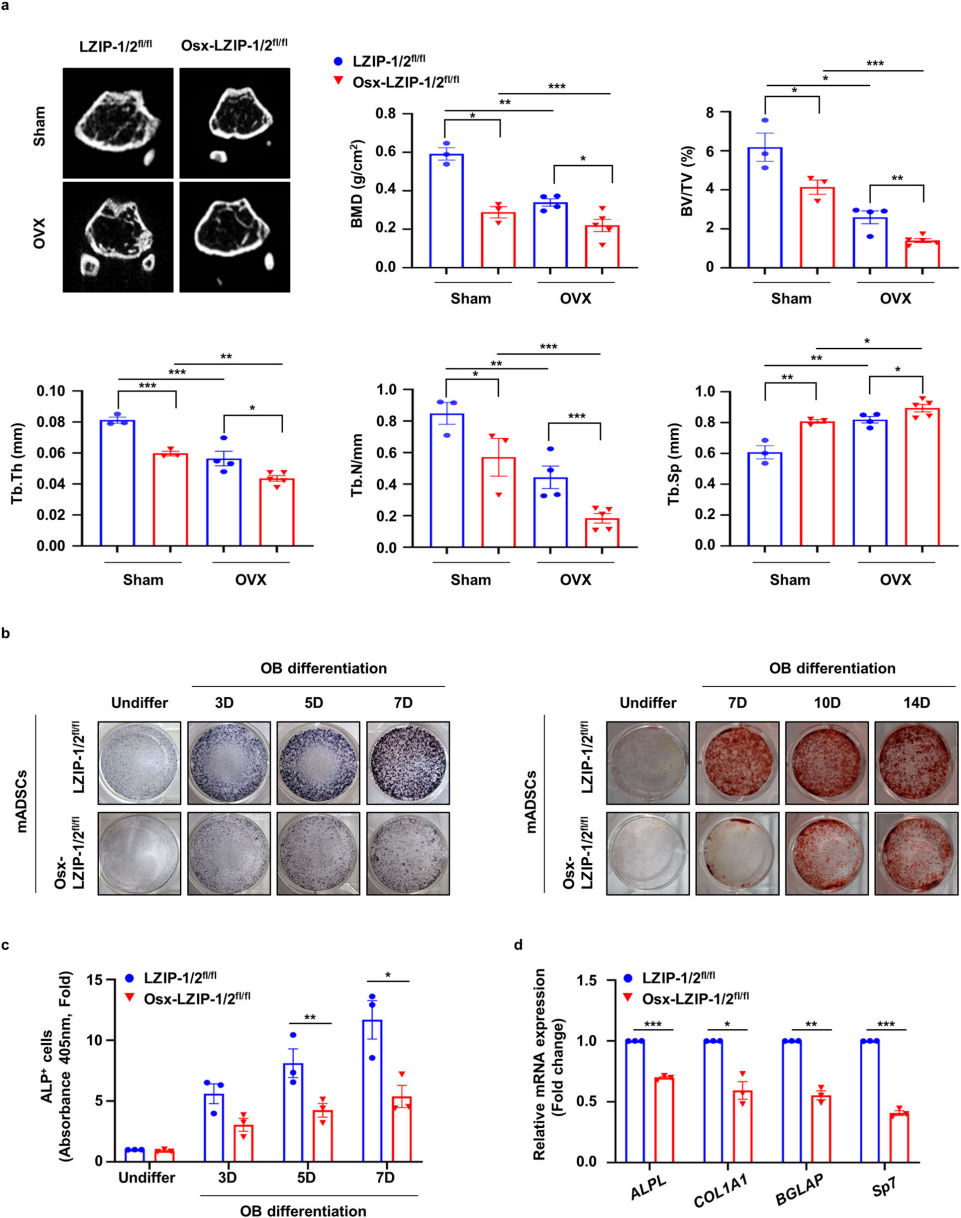
Deletion of sLZIP reduces bone mass and impedes osteoporosis recovery. **a**, μCT of trabecular bones in the femurs of LZIP-1/2^fl/fl^ (*n* = 3–4) and Osx-LZIP-1/2^fl/fl^ (*n* = 3–5) mice was performed. Representative μCT 2D images are shown. BMD, BV/TV, Tb.Th, Tb.N and Tb.Sp were quantified via μCT image analysis. **b**, For ALP staining, ADS cells derived from LZIP-1/2^fl/fl^ and Osx-LZIP-1/2^fl/fl^ mice were seeded at a density of 2 × 10^5^ cells/well and differentiated into OBs for 3, 5 and 7 days (D) in osteogenic medium. For ARS staining, ADS cells isolated from LZIP-1/2^fl/fl^ and Osx-LZIP-1/2^fl/fl^ mice were differentiated into OBs for the indicated times. The cells were stained with 2% ARS solution. **c**, ADS cells derived from LZIP-1/2^flfl^ and Osx-LZIP-1/2^fl/fl^ mice were seeded into 96-well plates at a density of 4 × 10^4^ cells/well. ALP activity was quantified via an ALP assay kit. **d**, ADS cells derived from LZIP-1/2^fl/fl^ and Osx-LZIP-1/2^fl/fl^ mice were differentiated into OBs for 7 days in osteogenic medium. The mRNA expression level was determined via qRT‒PCR, which was performed in triplicate for each group. The mRNA levels of OB differentiation markers were determined via qRT‒PCR, which was performed in triplicate for each group. Error bars show the mean ± s.e.m. *P < 0.05, **P < 0.01 and ***P < 0.001 (unpaired, two-tailed Student’s *t*-test).

Bone marrow-derived MS cells are multipotent stromal cells that differentiate into various lineages. *Sp7* (Osx) is a specific target of MS cells^22^. To investigate the role of sLZIP in bone regeneration in osteoporosis, we generated MS cell-specific sLZIP conditional knockout (KO) mice by crossing Osx-cre and murine LZIP-1/2^fl/fl^ mice and determined the mRNA expression of murine LZIP-1/2 in several tissues. Compared with control LZIP-1/2^fl/fl^ mice, sLZIP KO (Osx-LZIP-1/2^fl/fl^) mice presented decreased mRNA expression of murine LZIP-1/2 in MS cells (Supplementary Fig. 1b). The sLZIP KO mice in the OVX group presented reduced trabecular mass (Fig. 2a). Quantitative analyses revealed that BMD, BV/TV, Tb.Th, Tb.N and cortical thickness were lower in sLZIP KO mice than in LZIP-1/2^fl/fl^ mice, whereas Tb.Sp was greater in sLZIP KO mice than in LZIP-1/2^fl/fl^ mice (Fig. 2a and Supplementary Fig. 2a). We measured serum procollagen type I N-terminal propeptide (P1NP) levels. Serum P1NP levels were lower in sLZIP KO mice than in LZIP-1/2^fl/fl^ mice (Supplementary Fig. 2b). The results of ALP staining revealed that, compared with LZIP-1/2^fl/fl^ mice, sLZIP KO mice had reduced bone formation (Supplementary Fig. 2c). These results indicate that the deletion of sLZIP reduces bone mass and impedes recovery from osteoporosis. As sLZIP promoted bone formation in vivo, we investigated the effect of sLZIP on OB differentiation in vitro. ADS cells were isolated from the fat pads of sLZIP KO mice and differentiated into OBs. ALP staining, ALP activity analysis and ARS staining revealed that ADS cells from sLZIP KO mice presented decreased OB differentiation compared with ADS cells from LZIP-1/2^fl/fl^ mice (Fig. 2b, c). In addition, the mRNA expression levels of OB differentiation markers, including ALP (*ALPL*), osteocalcin (*BGLAP*), Osx (*Sp7*) and col1a1 (*COL1A1*), were lower in ADS cells from sLZIP KO mice than in those from LZIP-1/2^fl/fl^ mice (Fig. 2d). These results indicate that the deletion of sLZIP reduces bone mass and negatively affects recovery from osteoporosis.

### sLZIP induces bone fracture healing and regulates bone remodeling in osteoporosis

**Fig. 3:**
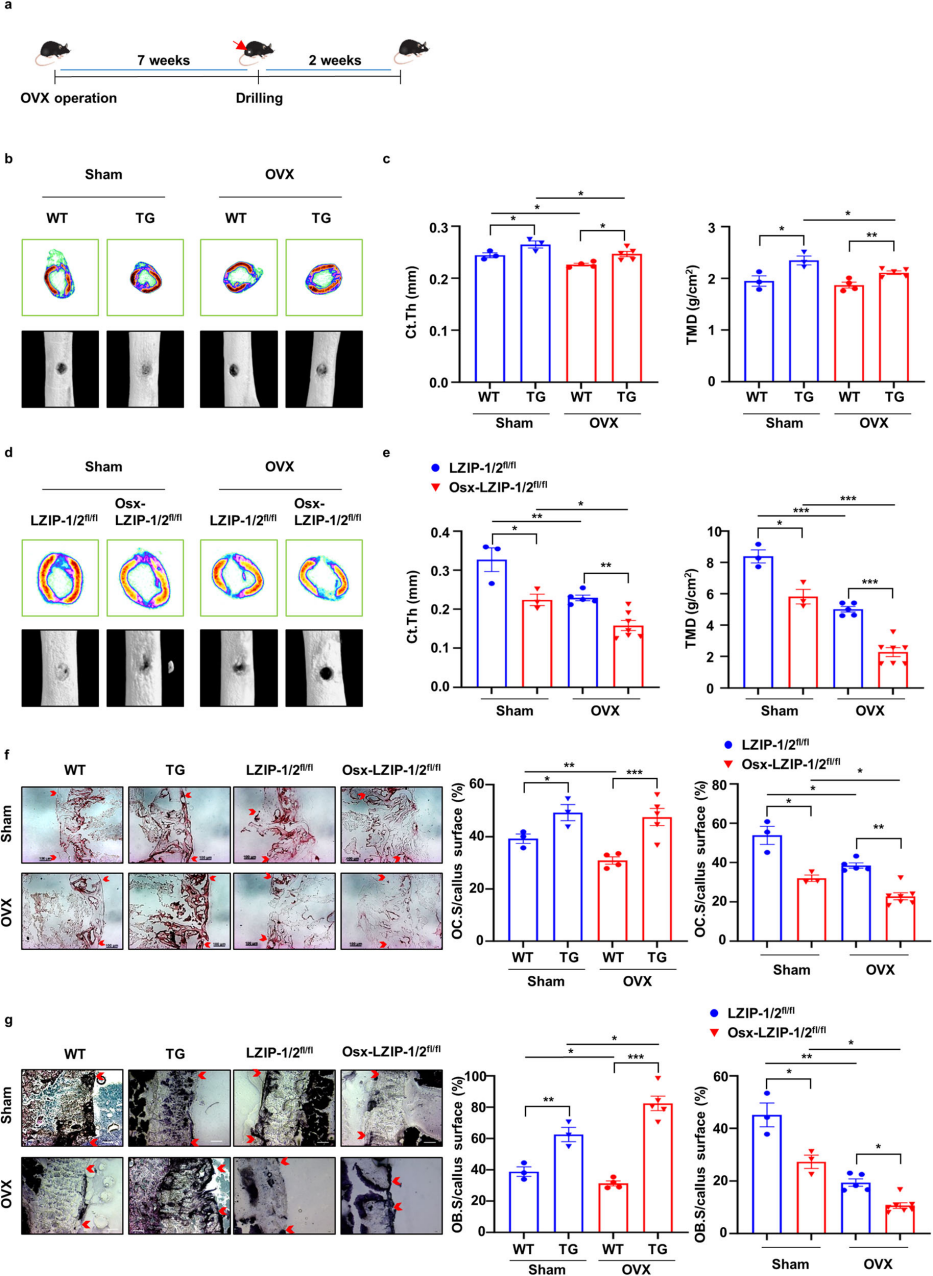
sLZIP induces bone fracture healing and regulates bone remodeling in osteoporosis. **a**, Eight-week-old mice were subjected to OVX surgery. Seven weeks after OVX surgery, the mice were anesthetized and underwent surgery to drill holes in the right femur. The mice that underwent drill-hole surgery were euthanized via cervical dislocation after 14 days. The right femur was harvested and fixed. **b**–**e**, Representative 2D and 3D images were obtained via μCT: representative 2D images were generated from μCT analysis after drill-hole surgery in WT and TG mice (**b**), Ct.Th and TMD were quantitatively analyzed using the μCT program (CTAn) (**c**), representative 2D images and 3D images were generated from μCT analysis after drill-hole surgery in the Osx-LZIP-1/2^fl/fl^ and LZIP-1/2^fl/fl^ mice (**d**) and Ct.Th and TMD were quantitatively analyzed using the μCT program (**e**). **f**, Mouse femurs were obtained after 2 weeks of decalcification and the defected region of the mouse femurs was cryosectioned (8 μm thickness). Decalcified femurs from female WT, sLZIP TG, LZIP-1/2^fl/fl^ and Osx-LZIP-1/2^fl/fl^ mice (*n* = 3–6) were subjected to TRAP staining. Representative images of callus sections demonstrating bone healing after drill-hole surgery. Scale bar, 100 μm. **g**, Mouse femurs were obtained in an undecalcified state and the defected region of the mouse femurs was cryosectioned (8 μm thickness). Undecalcified femurs from female WT, sLZIP TG, LZIP-1/2^fl/fl^ and Osx-LZIP-1/2^fl/fl^ mice (*n* = 3–6) were subjected to ALP staining. Representative images of callus sections demonstrating bone healing after drill-hole surgery. Scale bar, 100 μm. The ratio of the TRAP^+^ and ALP^+^ cell surface areas to the trabecular bone surface area was measured via ImageJ. Error bars show the mean ± s.e.m. *P < 0.05, **P < 0.01 and ***P < 0.001 (unpaired, two-tailed Student’s *t*-test).

As bone fractures frequently occur in osteoporosis^23^, we investigated whether sLZIP is involved in bone fracture healing. To examine the effects of sLZIP on bone fracture healing, we generated a bone fracture model by drilling a portion of the femur in osteoporotic mice. A drill-hole defect model was selected to simultaneously mimic bone fractures and avoid nonunion fractures^24^. Seven weeks after OVX surgery, holes were drilled in the right femurs of the mice. After 2 weeks, the femurs were collected (Fig. 3a). Quantitative analysis of bone fracture healing in the OVX group revealed that sLZIP TG mice presented faster callus formation and an increased volume of hard callus-containing bone in the defect region compared with those of WT mice (Fig. 3b). Compared with WT mice, sLZIP TG mice also presented increased cortical thickness and tissue mineral density in the defect region (Fig. 3c). However, compared with control mice, sLZIP KO mice presented decreased callus volume and formation in both the sham and OVX groups (Fig. 3d). In addition, the cortical thickness and tissue mineral density were lower in sLZIP KO mice than in control mice in both groups (Fig. 3e). These results indicate that sLZIP induces bone fracture healing and regulates bone remodeling in osteoporosis. Both OB activation and OC activation are involved in bone remodeling during fracture healing, with vital roles in callus and bone formation^25,26^. Therefore, we examined the levels of OBs and OCs on the surface of transiently formed calli at the site of a mouse femur defect during bone fracture healing. The results of TRAP staining revealed that, compared with WT mice, sLZIP TG mice presented increased TRAP^+^ cells on the callus surface in the defective region in both the OVX and sham groups (Fig. 3f). However, the number of activated OCs on the callus surface decreased in both groups of sLZIP KO mice (Fig. 3f). These results indicate that sLZIP increases bone resorption by inducing OC activation during bone healing. We investigated the effects of sLZIP on the activation of bone formation during fracture healing. To evaluate OB activation, mouse femurs were stained with an ALP staining solution. The results revealed that the number of ALP^+^ OBs was greater in sLZIP TG mice than in WT mice in the sham group, and sLZIP TG mice presented faster bone formation than WT mice in the OVX group (Fig. 3g). Compared with sLZIP TG mice, OVX sLZIP KO mice presented fewer ALP^+^ cells on the callus surface of the defective region in both groups, indicating that the deletion of sLZIP delayed bone fracture healing by inhibiting callus formation and reducing OC and OB activation in osteoporosis (Fig. 3g). Overall, these results suggest that sLZIP promotes bone resorption and formation during bone fracture healing.

### sLZIP functions as a modulator of the crosstalk between OBs and OCs

**Fig. 4:**
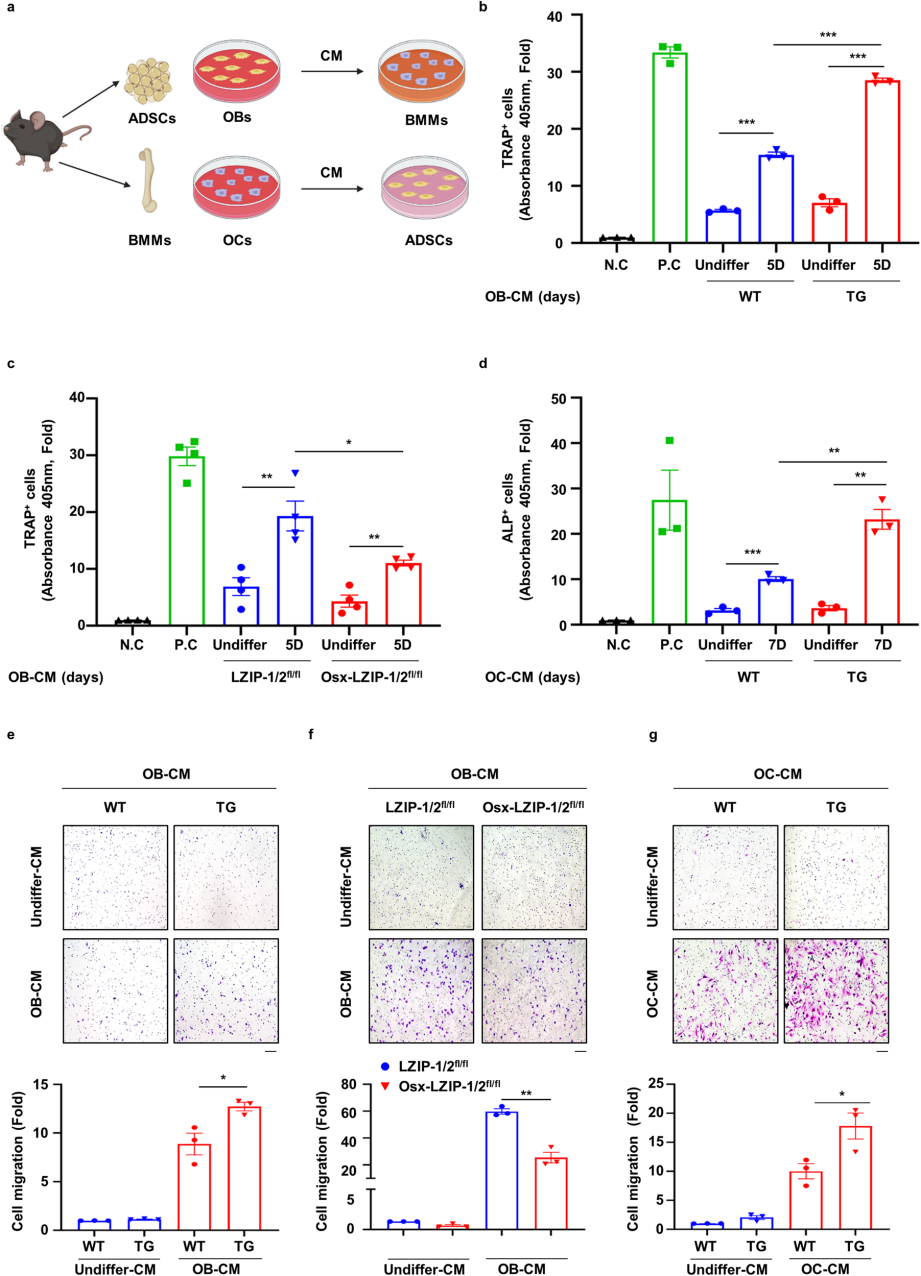
sLZIP functions as a modulator of the crosstalk between OBs and OCs. **a**, ADS cells isolated from mice were seeded into 12-well culture plates at a density of 8 × 10^4^ cells/well and differentiated into mature OBs for 5 and 7 days. CM was collected from mature OBs. BMMs isolated from mice were seeded into 12-well culture plates at a density of 2 × 10^5^ cells/well and differentiated into mature OBs for 7 days. CM was collected from mature OCs. **b**, **c**, BMMs isolated from WT mice were seeded at a density of 1 × 10^4^ cells/well in 96-well culture plates and cultured in α-MEM supplemented with OB-CM and 30 ng/ml M-CSF for 7 days. The cells were then subjected to TRAP staining. The negative control (N.C) cells were cultured in α-MEM supplemented with 30 ng/ml M-CSF for 7 days, and the positive control (P.C) cells were cultured in α-MEM supplemented with 30 ng/ml M-CSF and 50 ng/ml RANKL for 7 days. **d**, ADS cells isolated from WT mice were seeded into 96-well culture plates at a density of 1 × 10^4^ cells/well and exposed to OC-CM for 7 days. The cells were then subjected to ALP staining. **e**, **f**, CM from mature OBs was collected from mice. BMMs (8 × 10^4^ cells/well) derived from WT mice were plated on the inserts of transwell plates. For the migration assay, the cells were stained with 0.05% crystal violet. Representative images are shown (scale bar, 100 μm). The cells were counted from five randomly selected fields. **g**, CM from mature OCs was collected from mice. ADS cells (2 × 10^4^ cells/well) derived from WT mice were plated on the inserts of transwell plates. For the migration assay, the cells were stained with 0.05% crystal violet. Representative images are shown (scale bar, 100 μm). The cells were counted from five randomly selected fields. All the experiments were repeated three times independently. Error bars show the mean ± s.e.m. *P < 0.05, **P < 0.01 and ***P < 0.001 (unpaired, two-tailed Student’s *t*-test).

Bone remodeling, which plays a crucial role in bone repair, is a complex process regulated by crosstalk between bone formation and resorption^27^. As sLZIP participates in bone fracture healing and callus formation by activating both OBs and OCs, we postulated that sLZIP modulates bone remodeling by regulating the crosstalk between OBs and OCs. ADS cells and BMMs were isolated from mice and differentiated into OBs and OCs, after which OB-CM and OC-CM were collected and used to treat BMMs and ADS cells, respectively (Fig. 4a). TRAP staining and activity analysis revealed that OB-CM from sLZIP TG mice promoted osteoclastogenesis compared with that from WT mice (Fig. 4b and Supplementary Fig. 3a), whereas OB-CM from sLZIP KO mice decreased the population of TRAP^+^ cells compared with that from control mice (Fig. 4c and Supplementary Fig. 3b). We also examined the effect of sLZIP-mediated OC-CM on OB differentiation. ADS cells isolated from WT mice were treated with OC-CM obtained from sLZIP TG mice. Compared with OC-CM from WT mice, OC-CM from sLZIP TG mice presented increased ALP activity (Fig. 4d and Supplementary Fig. 3c). In addition, the mRNA expression of OB differentiation markers was greater in OC-CM from sLZIP TG mice than in that from WT mice (Supplementary Fig. 3d). These results indicate that sLZIP modulates OB–OC crosstalk. The migration of MS cells to the bone surface and pre-OCs to the injured region is crucial for bone remodeling and fracture healing^28,29^. Osteogenesis begins with MS cell recruitment at the site of bone remodeling, followed by cell proliferation^30^. As sLZIP regulates the crosstalk between OBs and OCs, we investigated whether sLZIP affects cell migration and proliferation during bone remodeling. The results of the transwell migration assay revealed that, compared with WT CM, OB-CM from sLZIP TG mice increased the migration of pre-OCs (Fig. 4e). In contrast, OB-CM from sLZIP KO mice induced decreased migration of pre-OCs compared with that from LZIP-1/2^fl/fl^ mice (Fig. 4f). However, the CM of undifferentiated OBs (undiffer-CM) from WT and sLZIP TG mice did not affect pre-OC migration (Fig. 4e, f). OB precursor cells were cocultured with OC-CM from WT and sLZIP TG mice to examine the effect of sLZIP on their migration. OC-CM from sLZIP TG mice induced greater migration of OB precursor cells compared with that from WT mice (Fig. 4g). Next, we investigated whether OC-CM from sLZIP TG mice affects the proliferation of OB precursor cells. However, we found no significant differences between OC-CM from WT and sLZIP TG mice (Supplementary Fig. 3e). These results indicated that sLZIP-induced CM increased cell migration during bone remodeling without affecting cell proliferation.

### sLZIP modulates bone remodeling by regulating the crosstalk between OBs and OCs

**Fig. 5:**
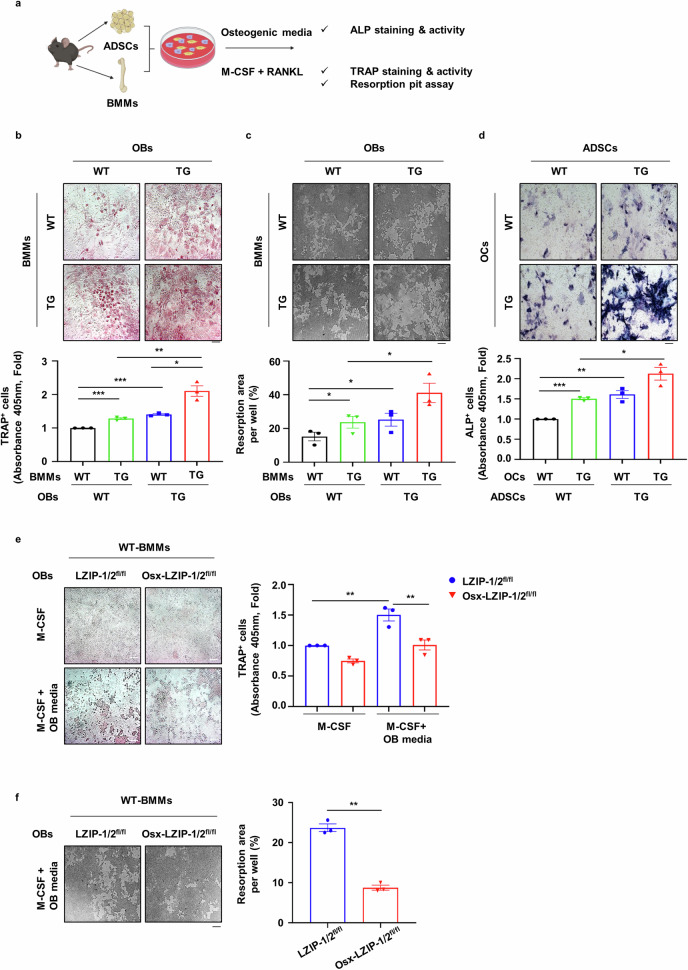
sLZIP modulates bone remodeling by regulating the crosstalk between OBs and OCs. **a**, ADS cells (ADSCs) and BMMs (1.5 × 10^4^ cells/well, ADS cells:BMMs at 1:1.5) were cocultured in 96-well culture plates. One day after seeding, the cells were cultured in osteogenic medium containing 30 ng/ml M-CSF or OC differentiation medium for 13 days. After differentiation, the cells were subjected to TRAP and ALP staining using the TRACP/ALP double-stain kit. **b**, BMMs and ADS cells were isolated from WT and sLZIP TG mice. The cells were subjected to TRAP staining using the TRACP stain kit. **c**, Ten days after culture in osteogenic medium containing 30 ng/ml M-CSF, the cells were detached with 5% bleach solution. The plates were washed with DDW and dried in a CO_2_ incubator. The resorbed area was calculated using ImageJ. Five fields from each well were randomly selected for quantification. **d**, BMMs and ADS cells were isolated from WT and sLZIP TG mice. An ALP staining kit was used for ALP staining. **e**, **f**, BMMs and ADS cells were isolated from LZIP-1/2^fl/fl^ and Osx-LZIP-1/2^fl/fl^ mice: a TRAP staining kit was used for TRAP staining of OCs in the coculture system and TRAP activity was measured at an absorbance of 405 nm (**e**) and the resorption area was calculated using ImageJ (**f**). All experiments were repeated three times independently. Error bars show the mean ± s.e.m. *P < 0.05, **P < 0.01 and ***P < 0.001 (unpaired, two-tailed Student’s *t*-test).

We further investigated the role of sLZIP in OB–OC crosstalk during bone remodeling via a coculture system of OBs and OCs. To examine the effect of sLZIP on mature OB-induced OC differentiation, OB precursor cells were cocultured with BMMs in osteogenic medium containing M-CSF (Fig. 5a). Compared with WT mouse-derived OBs, sLZIP TG mouse-derived OBs increased the population of TRAP^+^ cells when cocultured in osteogenic medium (Fig. 5b). OC differentiation was most effectively induced when pre-OCs and OBs derived from sLZIP TG mice were cocultured (Fig. 5b). Next, we performed a resorption pit assay to measure OC activity. Consistent with the TRAP staining results, compared with WT mouse-derived OBs, cocultures of WT mouse-derived BMMs and sLZIP TG mouse-derived OBs presented increased bone resorption (Fig. 5c). In particular, the coculture of BMMs and OBs from sLZIP TG mice potentiated bone resorption activity (Fig. 5c). To investigate OC-mediated osteogenesis, OB precursor cells were cocultured with BMMs in medium containing M-CSF and RANKL (Fig. 5a). Compared with that in WT mouse-derived OCs, the ALP^+^ cell population in sLZIP TG mouse-derived OCs was increased (Fig. 5d). The most notable increase in OB differentiation was observed when pre-OCs and OBs from sLZIP TG mice were cocultured (Fig. 5d). sLZIP KO mouse-derived OBs and WT mouse-derived BMMs were cocultured to examine the effects of sLZIP on osteoclastogenesis. OB-mediated osteoclastogenesis was alleviated in OBs derived from sLZIP KO but not in OBs derived from LZIP-1/2^fl/fl^ (Fig. 5e). Resorption activity was lower in the coculture of sLZIP KO mouse-derived OBs and WT mouse-derived BMMs than in the coculture of LZIP-1/2^fl/fl^ mouse-derived OBs and WT mouse-derived BMMs (Fig. 5f). These results suggest that sLZIP is involved in the coupling process and promotes bone remodeling via the regulation of OB–OC crosstalk.

### sLZIP regulates the crosstalk between OBs and OCs by inducing coupling factors

**Fig. 6:**
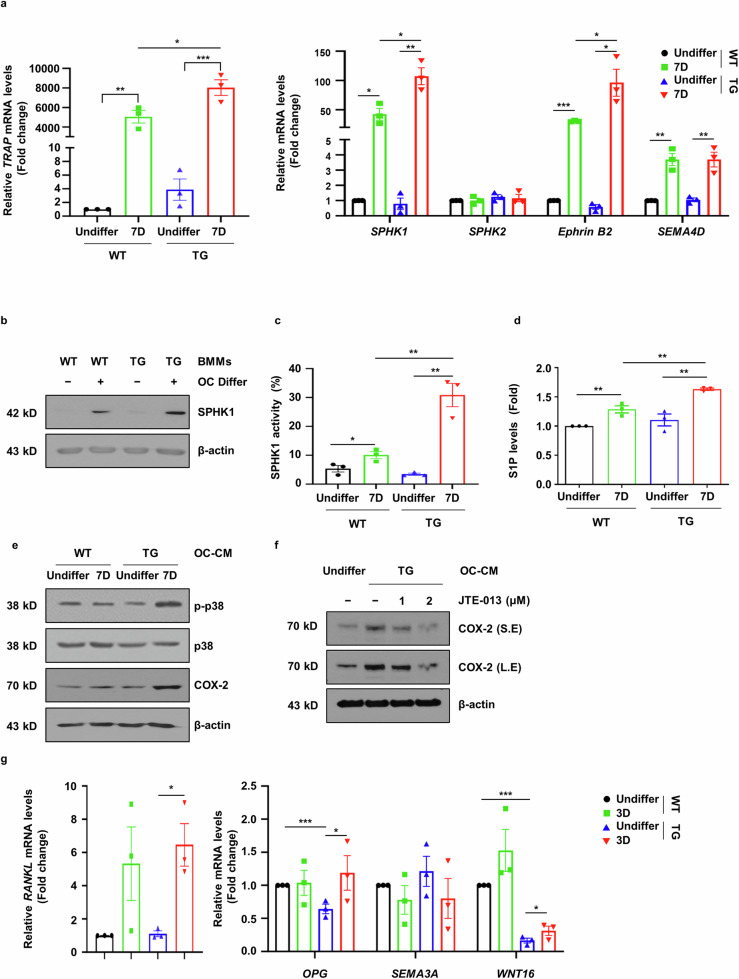
sLZIP regulates the crosstalk between OBs and OCs by inducing coupling factors. **a**–**d**, BMMs isolated from WT and sLZIP TG mice were seeded into 12-well culture plates at a density of 1.5 × 10^5^ cells/well and cultured for 7 days in α-MEM containing 50 ng/ml RANKL and 30 ng/ml M-CSF, then mRNA expression levels were determined via qRT‒PCR analysis with β-actin used as an internal control (**a**); protein expression levels were determined using western blotting (**b**); SPHK1 activity was determined in 30 μg of cell lysate (**c**) and S1P levels were analyzed via an ELISA kit (**d**). **e**, ADS cells derived from WT mice were exposed to differentiated OC-CM from WT and sLZIP TG mice. Protein expression levels were determined using western blotting. **f**, ADS cells were cultured with OC-CM from TG or JTE-013 cells (0, 1 or 2 μM) for 24 h. Protein levels were detected using western blotting. β-Actin was used as an internal control. **g**, ADS cells derived from WT and sLZIP TG mice were seeded into 12-well culture plates at a density of 8 × 10^4^ cells/well and differentiated into OBs for 3 days. The mRNA expression level was determined via qRT‒PCR analysis. All experiments were repeated three times independently. Error bars show the mean ± s.e.m. *P < 0.05, **P < 0.01 and ***P < 0.001 (unpaired, two-tailed Student’s *t*-test).

As sLZIP is involved in OB–OC crosstalk during bone remodeling, we investigated whether sLZIP affects coupling factor secretion. We examined the effect of sLZIP on the secretion of OC-mediated coupling factors during OC differentiation. S1P is synthesized through phosphorylation by SPHK1/2 and is involved in bone formation and survival^12,31^. Therefore, we examined the mRNA expression of SPHK1 during OC differentiation from progenitor cells. The mRNA level of SPHK1 was increased by sLZIP during OC differentiation, whereas the mRNA level of SPHK2 remained unaffected (Fig. 6a). Compared with WT mice, sLZIP TG mice presented increased SPHK1 protein expression during OC differentiation (Fig. 6b). To examine the effect of sLZIP on S1P synthesis by SPHK1, we performed SPHK1 activity assays. SPHK1 activity was greater in mature OCs derived from sLZIP TG mice than in those derived from WT mice (Fig. 6c). Compared with that in WT mice, the S1P level in mature OCs derived from sLZIP TG mice also increased by 13% (Fig. 6d). These results indicate that sLZIP induces S1P secretion by stimulating SPHK1 activity in OCs. S1P participates in bone formation by upregulating cyclooxygenase-2 (COX-2) via the p38/ERK pathway^31^. We investigated whether sLZIP-induced S1P affects the activation of the p38/ERK pathway in ADS cells. OC-CM derived from sLZIP TG mice induced the phosphorylation of p38 and increased COX-2 expression compared with that in WT mice (Fig. 6e). To confirm whether sLZIP-induced S1P was involved in COX-2-mediated bone formation, we used the S1P receptor inhibitor JTE-013. OC-CM derived from sLZIP TG mice presented increased COX-2 expression compared with that in undifferentiated mice; however, COX-2 expression was decreased by JTE-013 in a dose-dependent manner (Fig. 6f). We also examined the effects of sLZIP on the OB-mediated secretion of coupling factors during OB differentiation. *WNT16* mRNA expression was lower in OBs from sLZIP TG mice than in those from WT mice (Fig. 6g). These results indicate that sLZIP regulates crosstalk between OBs and OCs by inducing the secretion of coupling factors.

### sLZIP-overexpressing ADS cells promote bone formation and bone repair in osteoporosis

**Fig. 7:**
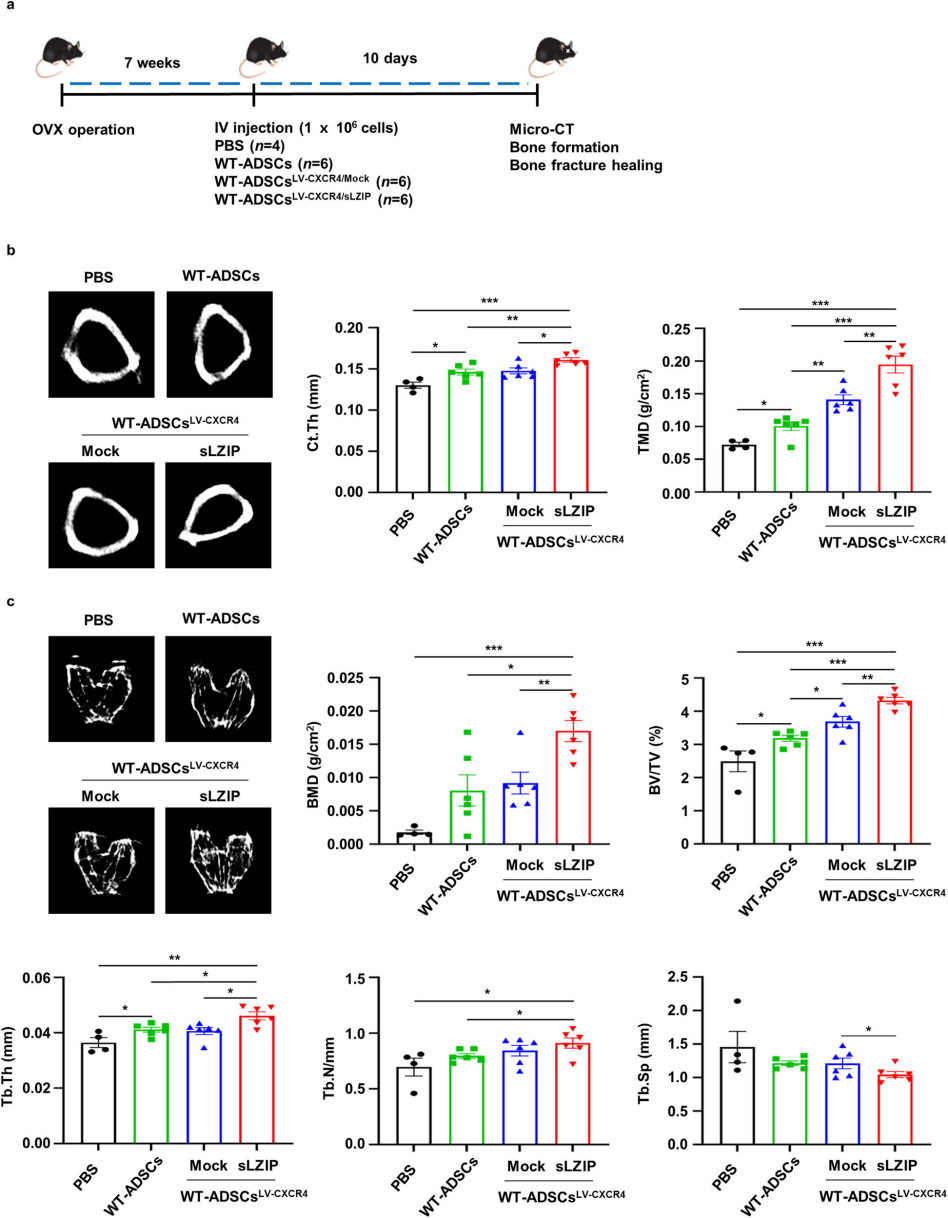
sLZIP-overexpressing ADS cells promote bone formation in osteoporosis. **a**, OVX surgery was performed on WT mice. After 7 weeks, each type of ADS cell (1 × 10^6^ cells/100 μl of PBS) was intravenously (IV) injected into OVX mice. After 10 days, mouse femurs were analyzed using μCT. **b**, Representative 2D images were obtained via μCT analysis. Quantification of Ct.Th and TMD were analyzed using the μCT program. **c**, Representative 2D images were generated from μCT analysis. BMD, BV/TV, Tb.Th, Tb.N and Tb.Sp were quantified using μCT. Error bars show the means ± s.e.m. *P < 0.05, **P < 0.01 and ***P < 0.001 (unpaired, two-tailed Student’s *t*-test).

**Fig. 8:**
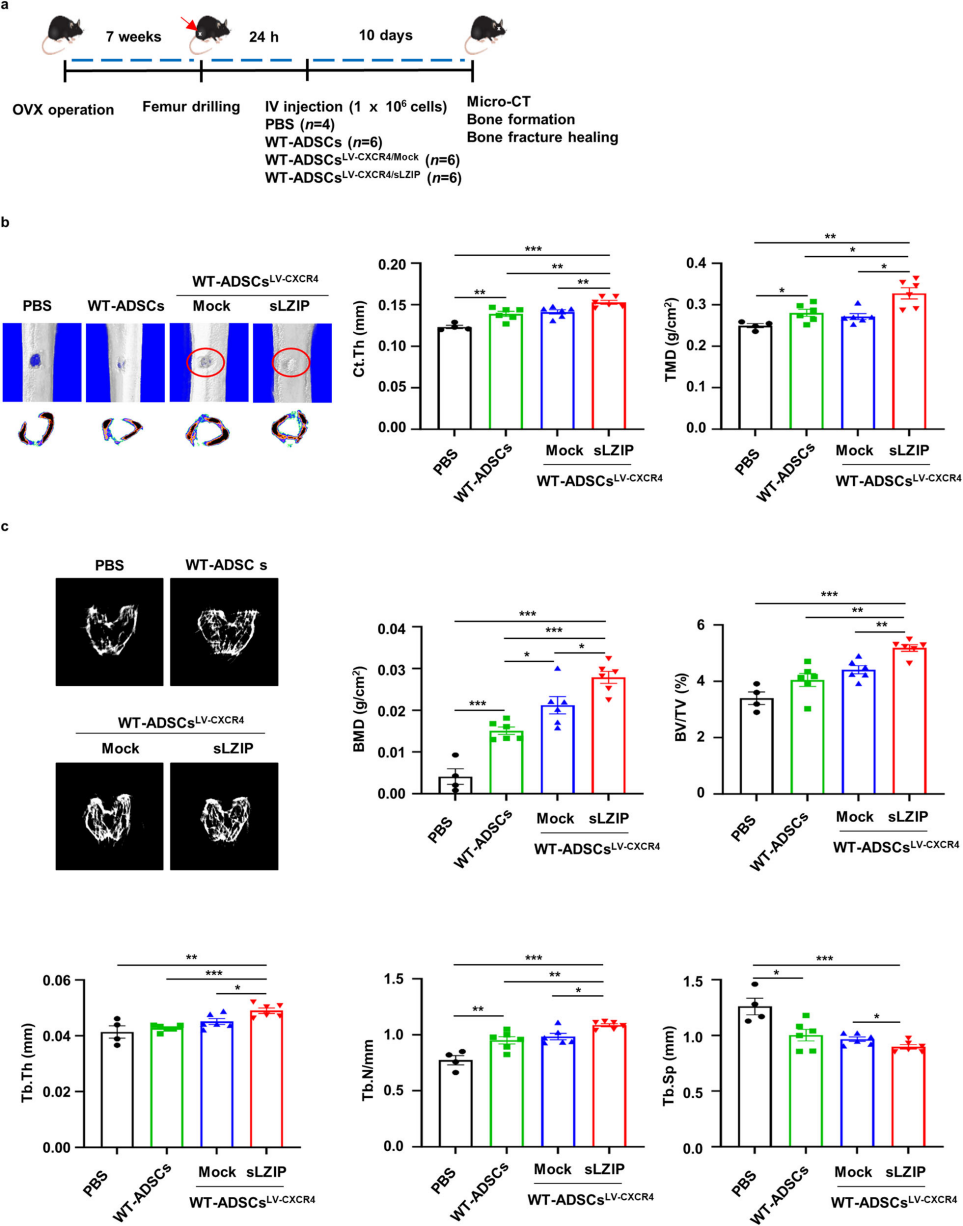
sLZIP-overexpressing ADS cells promote bone repair in osteoporosis. **a**, Eight-week-old mice were used for OVX surgery. Seven weeks after surgery, the mice were anesthetized and underwent surgery to drill holes in the right femur. After 24 h, each type of ADS cell (1 × 10^6^ cells/100 μl of PBS) was intravenously (IV) injected into the mice. After 10 days, mouse femurs were analyzed using μCT. **b**, Representative 2D and 3D images were generated from μCT analysis. Quantification of Ct.Th and TMD were analyzed using the μCT program. **c**, Representative 2D images were generated from μCT analysis. BMD, BV/TV, Tb.Th, Tb.N and Tb.Sp were quantified using the μCT program. Error bars show the means ± s.e.m. *P < 0.05, **P < 0.01 and ***P < 0.001 (unpaired, two-tailed Student’s *t*-test).

We investigated whether sLZIP could be used in cell therapy to treat osteoporosis. To examine the effect of ADS cells isolated from sLZIP TG mice (sLZIP TG-ADS cells) on osteoporosis, we performed a drilling assay on the right femurs of WT mice and then administered each type of ADS cell via intravenous injection to each group (Supplementary Fig. 4a). The results revealed that cortical thickness (Ct.Th) and tissue mineral density (TMD) were greater in sLZIP TG-ADS cells than in WT-ADS cells (Supplementary Fig. 4b). Compared with WT-ADS cells, sLZIP TG-ADS cells presented increased BMD, BV/TV and Tb.Th values (Supplementary Fig. 4c). These results indicate that sLZIP TG-ADS cells have potential as a cell therapy for bone fractures and reduced bone mass. The C-X-C motif receptor 4 (CXCR4) plays a role in MS cell migration to the bone marrow, demonstrating its potential as a therapeutic agent for osteoporosis^32^. To optimize the efficiency of cell therapy, we generated lentiviruses overexpressing CXCR4 and sLZIP (LV-CXCR4/sLZIP) and infected them with ADS cells isolated from WT mice. Seven weeks after OVX surgery, each group was injected with PBS, WT-ADS cells or LV-CXCR4/sLZIP-ADS cells. After 10 days, bone formation was analyzed using μCT (Fig. 7a). Compared with those of the LV-CXCR4/mock-ADS cells, the Ct.Th, TMD, BMD, BV/TV and Tb.Th of the LV-CXCR4/sLZIP-ADS cells were greater, whereas the Tb.Sp was lower (Fig. 7b, c). To examine the effect of LV-CXCR4/sLZIP-ADS cells on bone fracture healing, we performed a drilling assay on the right femurs of mice after OVX surgery; four groups were injected with PBS, WT-ADS cells or virus-infected ADS cells (Fig. 8a). The 3D images revealed that, compared with those in the PBS group, the bone recovery in the defect areas of the mice injected with WT-ADS cells or LV-CXCR4/mock-ADS cells was greater (Fig. 8b). Compared with the WT-ADS cell and LV-CXCR4/mock-ADS cell groups, the LV-CXCR4/sLZIP-ADS cell group exhibited more effective bone repair (Fig. 8b). The results of μCT revealed that, compared with PBS, WT-ADS cells and LV-CXCR4/mock-ADS cells presented increased Ct.Th and TMD values, along with elevated BMD and Tb.N values (Fig. 8b, c). These results showed that, compared with control mice, mice injected with LV-CXCR4/sLZIP-ADS cells presented increased bone formation and fracture recovery. These findings indicate that sLZIP-overexpressing ADS cells promote bone formation and repair in osteoporosis and have the greatest ability to treat osteoporosis and fracture recovery.

## Discussion

Substantial progress has been made in the development of novel treatment strategies for osteoporosis, but these medicines have many adverse effects and no cure has been achieved^33^. Therefore, understanding the molecular mechanisms underlying osteoporosis treatment is important. Osteoporosis is a common bone remodeling disorder^34^. Bone remodeling is performed by a functional and anatomic structure known as the basic multicellular unit (BMU). The BMU is the anatomical structure produced by new bone filling by OBs in the space absorbed by the OCs^35^. Previous studies suggest that sLZIP not only regulates the balance between adipogenesis and osteogenesis but also induces the differentiation of BMMs into OCs by promoting NFATc1 transactivation^20,21^. As sLZIP functions as a modulator of both bone resorption and formation, we speculated that sLZIP is an essential factor for regulating BMU and bone remodeling.

Compared with control mice in the OVX group, sLZIP KO mice presented reduced bone mass, whereas the bone mass of OVX sLZIP TG mice was restored to that of sham WT mice. These results indicate that sLZIP prevents bone loss in patients with osteoporosis. Osteoporosis with attenuated bone mass is followed by bone porosity and fragility fractures^2,36–38^. Therefore, we investigated the effect of sLZIP on the healing of osteoporosis-induced bone fractures. Compared with control mice in the OVX group, sLZIP KO mice presented reduced bone repair. We confirmed that bone mass was reduced by OVX. However, OC formation was lower in the OVX group than in the sham group. These results can be explained by clinical surveys showing that osteoporotic patients have delayed fracture healing compared with non-osteoporotic patients when fractures occur^39^. Several studies have reported on this topic. PDK1 deletion in OCs delays fracture union and repair by reducing callus resorption^40^. Compared with those in sham mice, the bone fracture model in OVX mice resulted in decreased callus formation and BMD^24^. OCs are responsible for removing soft calluses and forming hard calluses during the bone fracture healing process^41^. Therefore, the reduced OC content on the callus surface in OVX mice delayed callus formation caused by osteoporosis and decreased OC activity. Typically, both anabolic and anti-bone-resorptive osteoporosis treatments act through a single pathway^33^. Denosumab, a monoclonal antibody against RANKL, also inhibits OC activity^42^. As one-pathway functional drugs have many side effects, dual-function drugs that target both mechanisms may have superior therapeutic effects. However, no such drugs are currently available. Therefore, alternative treatments for osteoporosis are urgently needed.

MS cells are undifferentiated cells with self-renewal and multilineage differentiation abilities and are closely related to the progression of osteoporosis^43^. Accordingly, MS cells have been studied over the past few decades for their potential to establish therapeutic strategies for various pathophysiological dysfunctions in degenerative medicine^44^. MS cells have promising applications in the treatment of osteoporosis. The direct transplantation of MS cells into OVX mice promotes osteogenic differentiation^45^. When bone marrow-derived MS cells were injected into OVX mice via the tail vein, the MS cell-transplanted OVX mice presented significantly increased BMD, Tb.N, Tb.th and BV values^45^. Homing is the first phase of bone repair, in which MS cells migrate to the bone marrow to fulfill local functions and aid in recovery^32^. CXCR4 participates in homing MS cells to the bone marrow^32^. Compared with sham mice, OVX mice injected with MS cells cotransfected with RANK-Fc and CXCR4 presented increased BMD^46^. However, MS cell transplantation also has side effects caused by excessive bone formation. Therefore, studies on dual-function drugs using CXCR4-MS cells are needed.

Our results indicate that sLZIP accelerates bone fracture healing by promoting osteoclastogenesis and osteoblastogenesis. OB-CM derived from sLZIP KO mice shows reduced BMM migration and OC differentiation. OC-CM derived from sLZIP KO mice presented decreased OB precursor cell migration and differentiation. The results of the coculture study revealed that sLZIP regulates bone remodeling by controlling the crosstalk between OBs and OCs. These results suggest that sLZIP is a suitable target for controlling both pathways and can provide a molecular mechanism for the development of novel cell therapies. Furthermore, we found that, compared with WT-ADS cells, LV-CXCR4/sLZIP-ADS cells demonstrated a therapeutic effect by increasing bone formation in osteoporotic mice.

The coupling process is vital in bone diseases because pathological bone diseases are caused by dysregulation of the interplay between OBs and OCs^34^. Therefore, studies on novel coupling factors are important for osteoporosis treatment, and many studies have been conducted on the coupling factors involved in bone remodeling. S1P is synthesized by SPHK1/2 in OCs and secreted to induce bone formation and survival by binding to S1PR, an S1P receptor in the membrane of OBs^12^. OC-derived semaphorin 4D binds to plexin-B1 on the surface of OBs and inhibits OB differentiation^47^. Recent studies have reported factors that regulate coupling factors. TGIF1 promotes osteoclastogenesis in OBs by inhibiting semaphorin 3E, an OB-derived coupling factor that suppresses OC differentiation^48^. Although S1P secreted from OCs acts as a coupling factor for OB formation, the molecular mechanisms underlying S1P regulation in OCs remain unclear.

In this study, we demonstrated that sLZIP enhances S1P secretion by inducing SPHK1 expression during OC differentiation and that sLZIP-secreted S1P in OCs affects OB differentiation. These results suggest that sLZIP is essential for regulating S1P in OCs and that sLZIP acts as an important regulator of coupling factors. OB-derived WNT16 reduces osteoclastogenesis by interfering with RANK signaling^38^. We found that sLZIP TG reduced *WNT16* mRNA expression during OB differentiation. These results indicate that sLZIP regulates osteoclastogenesis by inhibiting WNT16 secretion by OBs. Therefore, our findings demonstrated that sLZIP plays an important role as a modulator of coupling factor secretion in both OCs and OBs.

Coupling factors not only act as secreted proteins but also mediate direct cell–cell communication between OBs and OCs. The Ephrin B2 ligand within the OC membrane interacts with EphB4, which is located on the OB membrane. The Ephrin B2/EphB4 axis affects both OBs and OCs. Claudin 11 maintains bone homeostasis via bidirectional Ephrin B2/EphB4 signaling^49^. We found that the mRNA levels of Ephrin B2 were increased by sLZIP during differentiation into mature OCs. However, Ephrin B2 deletion in BMMs does not result in any bone abnormalities^50^. As bone mass is regulated by sLZIP, Ephrin B2 does not contribute to the sLZIP-induced coupling process.

Bone remodeling is a complex process in which various cells interact simultaneously in localized spaces. Drugs currently used to treat osteoporosis disrupt bone homeostasis owing to their excessive absorption and bone formation. Therefore, the key to the treatment of osteoporosis is to reduce the side effects of drugs by targeting two pathways rather than one. In this study, we demonstrated that sLZIP exerts an osteoprotective effect by promoting bone resorption and formation and functions effectively in restoring bone fractures caused by osteoporosis. This study provides a molecular basis for developing treatments to control bone absorption and formation. Thus, sLZIP is a dual-function medicinal candidate for the treatment of metabolic bone diseases.

## Supplementary information

Supplementary Information.

## Data Availability

The data underlying this article will be shared upon reasonable request to the corresponding author.
