# Supplementary material for: sLZIP functions as a key modulator of bone remodeling by regulating the crosstalk between osteoblasts and osteoclasts
Source: Exp Mol Med. 2025 Mar 3;57(3):601–15. doi: 10.1038/s12276-025-01414-3 (PMC11958637; doi:10.1038/s12276-025-01414-3)
Supplement: Supplementary file 1 — Supplementary Information. [file 12276_2025_1414_MOESM1_ESM.docx]

**Materials**

Dulbecco’s modified Eagle’s medium (DMEM) was purchased from Thermo Fisher Scientific (Waltham, MA). Eagle’s minimum essential medium, alpha modification (α-MEM), and fetal bovine serum (FBS) were obtained from GE Healthcare Life Sciences (Pittsburg, PA, USA). Recombinant human/mouse/rat bone morphogenetic protein 2 (BMP-2) was obtained from R&D systems, Inc (McKinley Place, NE, USA). L-ascorbic acid, dexamethasone, and β-glycerophosphate were purchased from Millipore (Burlington, MA, USA). Mouse M-CSF was purchased from PeproTech (Rocky Hill, NJ, USA). Mouse RANKL was obtained from R&D Systems, Inc. Type I collagenase was purchased from Millipore (Billerica, MA). The fluorescent mounting medium was obtained from Dako (Santa Clara, CA, USA). Tissue-Tek^®^ O.C.T compound was purchased from Sakura Finetek (Torrance, CA, USA). Antibodies against SPHK1 (G-11) and COX-2 were purchased from Santa Cruz Biotechnology (Dallas, TX, USA).

**Ethics statement for animal study**

We adhered to the essential procedures specified in the Animal Research: Reporting of In Vivo Experiments (ARRIVE) guidelines to minimize suffering for the experimental animals and ensure appropriate care and welfare. The animal studies were approved by the Ethics Committee of Animal Experiments at Korea University (KUIACUC-2021-0095), and all procedures were performed in strict accordance with the Korea University Guide for the Care and Use of Animals in Laboratory Experiments. The mouse strain C57BL6 were used for the animal study. Mice were purchased from Gyerim experimental animal resource center (Seoul, South Korea) and maintained at 22 ± 2 °C and 50 ± 10% humidity under a 12 h light-dark regimen. In all animal studies, mice were anesthetized with isoflurane following the AVMA (American Veterinary Medical Association) guidelines for abdominal laparotomy. Animals were euthanized in strict accordance with ethical guidelines. CO_2_ was injected into the chamber at a rate of 30–70% charging per minute. After visual confirmation of death, complete euthanasia was induced by exposing the mice to CO_2_ for an additional minute and death was confirmed by monitoring their heartbeats.

**Isolation of primary mouse ADSCs**

Primary mouse ADSCs were isolated as previously described^1^. Briefly, 8-week-old mice were euthanized by cervical dislocation. After sterilization with 70% ethanol, the abdominal cavity was opened and the desired fat pad was removed. Fat was incubated with collagenase type I buffer for 60 min. After collagenase digestion, the samples were centrifuged at 300 × g for 5 min at 25 °C. Pellets were washed with 1% BSA and centrifuged at 300 × g for 5 min. Collected ADSCs were resuspended in DMEM containing 10% FBS, 100 U/mL penicillin, and 100 μg/mL streptomycin. Cells were plated on a culture dish and cultured in a 37 °C humidified incubator containing 5% CO_2_. The medium was changed every 2 days until the cells reached 80% confluence.

**Isolation of primary mouse BMMs**

Mice aged 8–12 weeks were euthanized by cervical dislocation. After sterilization with 70% ethanol, the skin near the femur was clipped outward to expose the hind legs. All muscle tissues were removed from the isolated femurs and tibias. Superior and inferior ends of the isolated bones were cut, and bone marrow cells were flushed out with α-MEM containing 10% FBS, 100 U/mL penicillin, and 100 μg/mL streptomycin using a 10 mL syringe and 24-gauge needle. A single-cell suspension was created by pipetting, and cells were passed through a cell strainer with a 100 μm pore size (SPL Life Science Co., Pocheon, South Korea) to remove other bone components. After centrifugation at 200 × g for 3 min at 25 °C, cells were resuspended in fresh medium and cultured at 37 °C in a humidified incubator containing 5% CO_2_.

**Measurement of procollagen type I N-terminal propeptide (P1NP)**

Mouse sera from 8-week-old mice were collected and serum P1NP was measured using an ELISA kit (Elabscience Biotechnology, Wuhan, China) according to the manufacturer’s instructions.

**REFERENCES**

1. Yu G, Wu X, Kilroy G, Halvorsen YD, Gimble JM, Floyd ZE. Isolation of murine adipose-derived stem cells. *Methods Mol Biol*, **702:** 29-36 (2011).


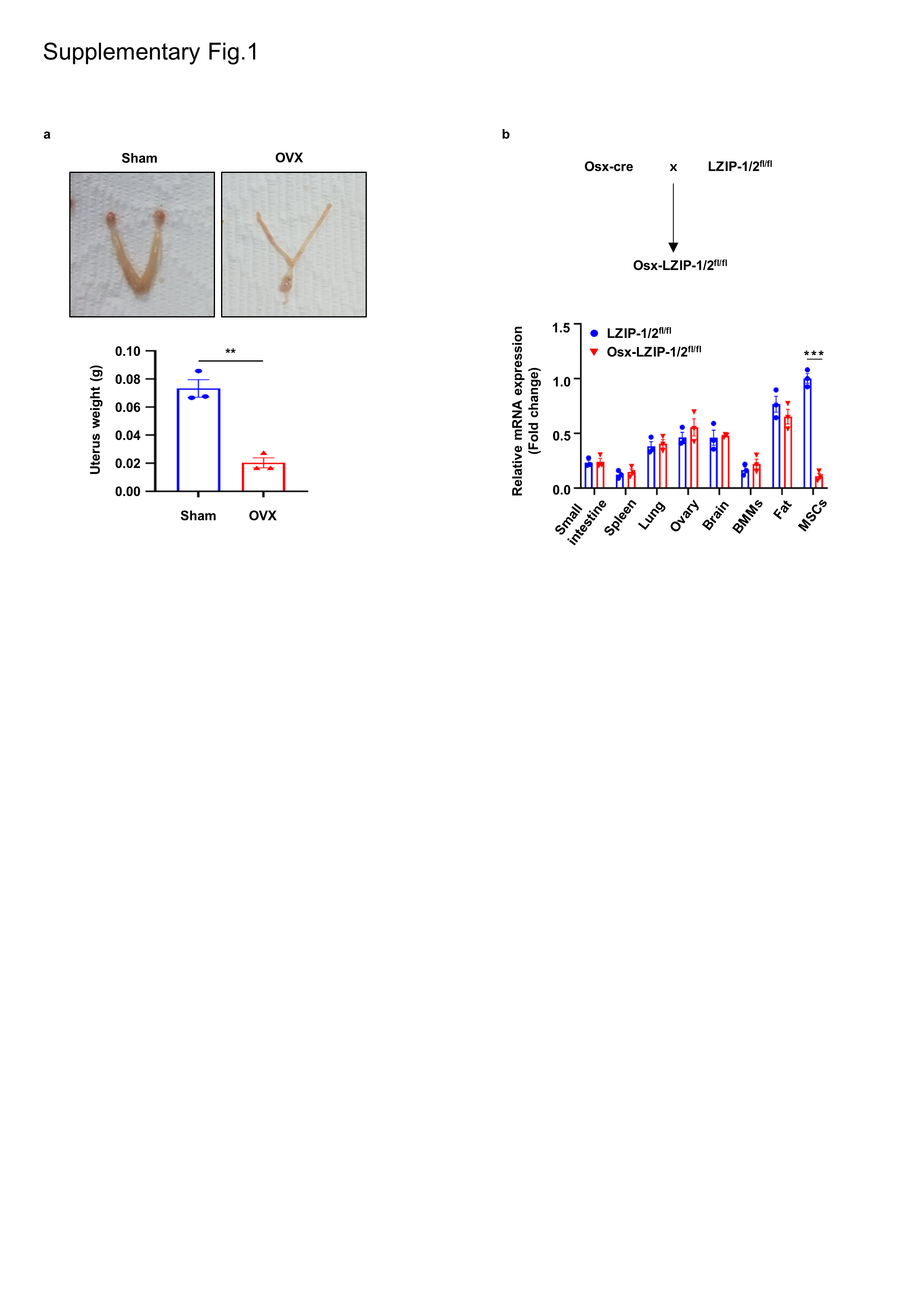


**Supplementary Fig. 1. Generation of OVX mouse models and Osx-LZIP-1/2^fl/fl^ mice. a** The uteri were harvested seven weeks after OVX surgery and weighed. **b** Osx-LZIP-1/2^fl/fl^ mice were generated by crossing Osx-cre mice and LZIP-1/2^fl/fl^ mice and tissues were isolated. The mRNA expression of murine LZIP-1/2 was determined by qRT-PCR. Error bars: mean ± SEM., ***p* < 0.01 , ****p* < 0.001 (unpaired, two-tailed student’s *t*-test).


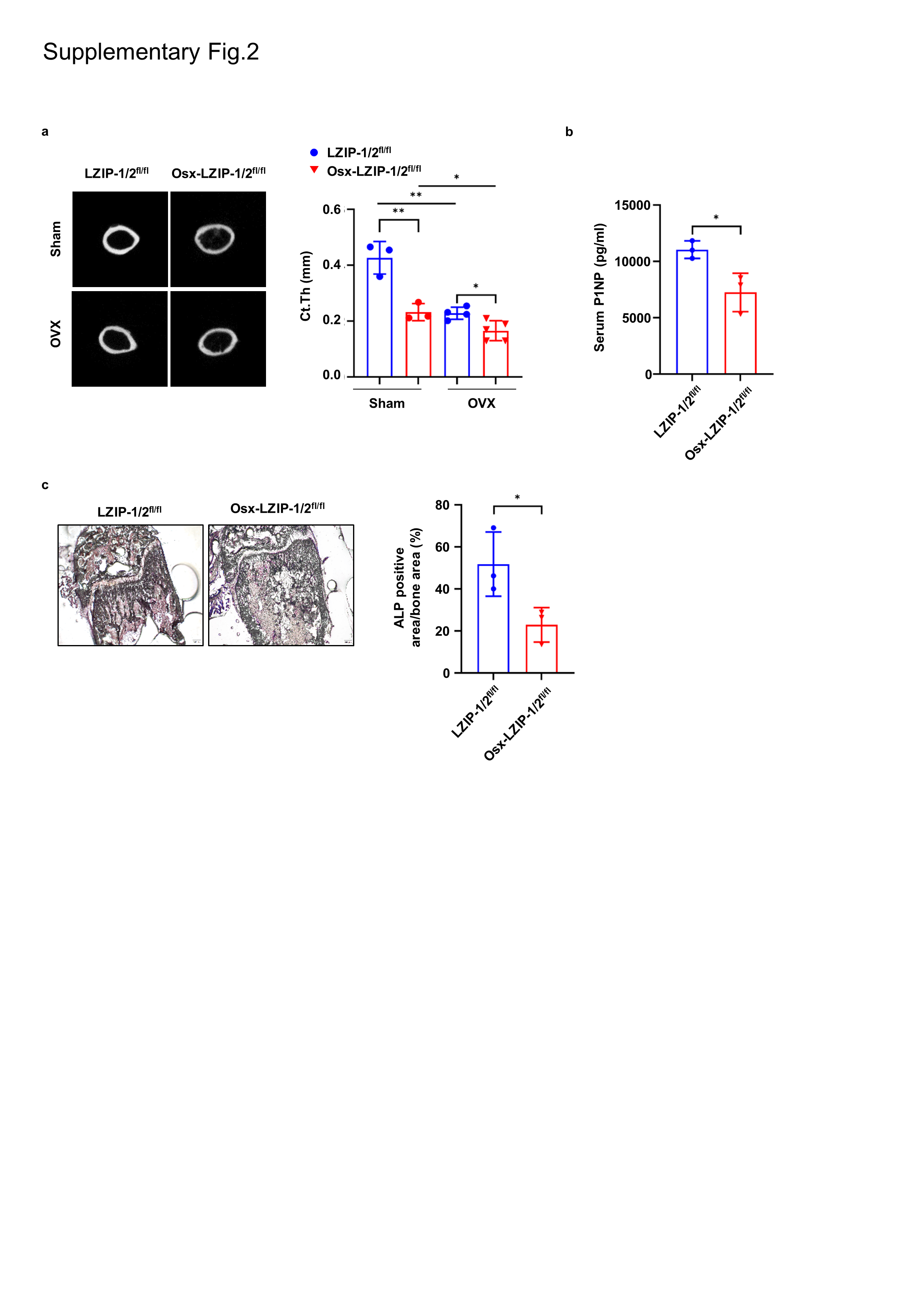


**Supplementary Fig. 2. sLZIP KO mice reduced bone formation. a** Representative 2D images were generated from μCT analysis. Quantification of cortical thickness (Ct.Th) were analyzed using the μCT program (CTAn). **b** Sera were collected from Osx-LZIP-1/2^fl/fl^ (*n* = 3) and LZIP-1/2^fl/fl^ mice (*n* = 3). **c** Samples were cryo-sectioned into 10 μm-thick slices without decalcification. The samples were subjected to ALP staining and then measured using the ImageJ program. Error bars: mean ± SEM., ***p* < 0.01 , ***p* < 0.01 (unpaired, two-tailed student’s *t*-test).


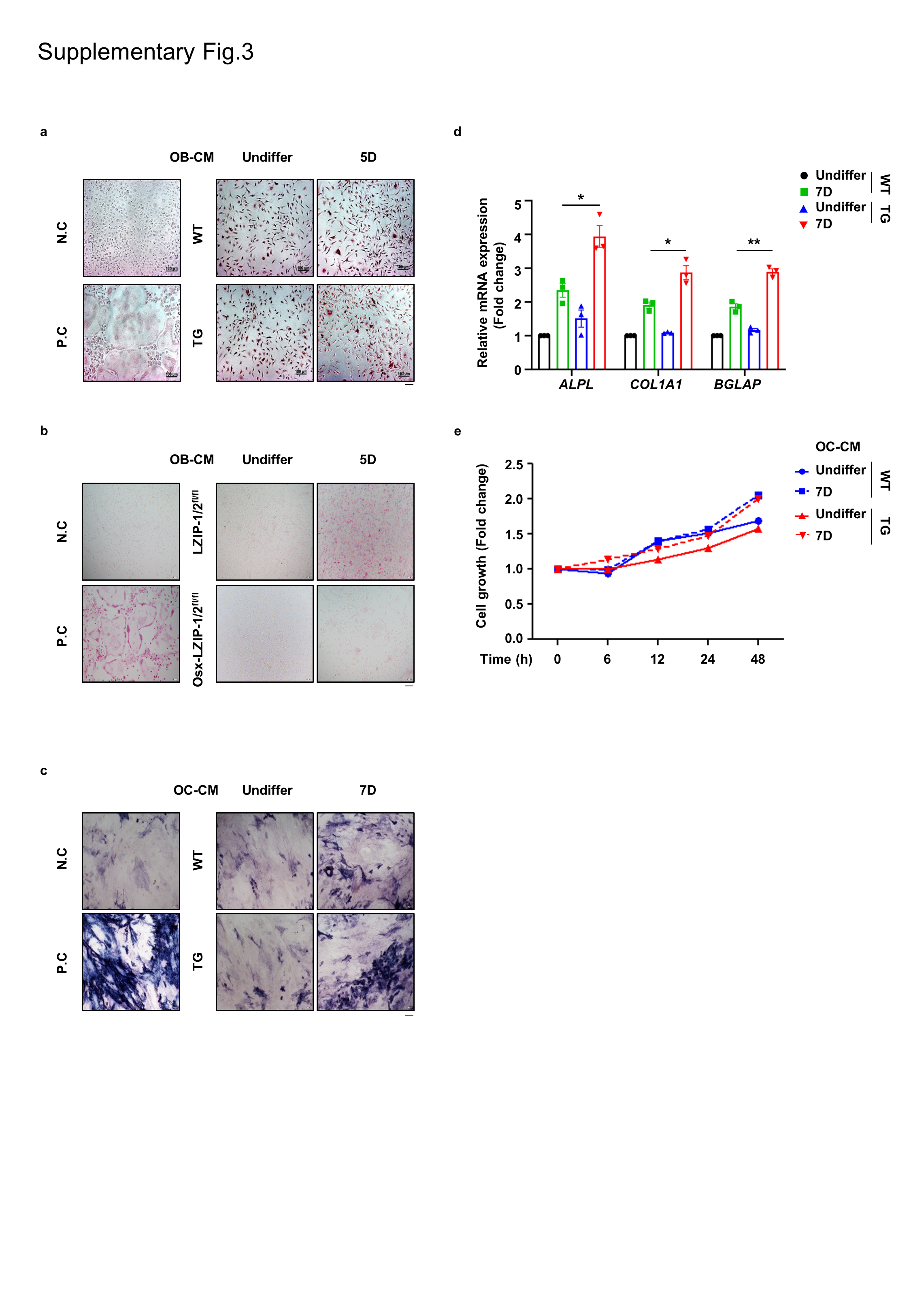


**Supplementary Fig. 3.** **sLZIP functions as a modulator of the crosstalk between OBs and OCs. a-e** ADSCs isolated from mice were seeded at a density of 8 × 10^4^ cells/well in 12-well culture plates and differentiated into mature OBs for 5 days. CM was collected from mature OBs. BMMs isolated from mice were seeded at a density of 2 × 10^5^ cells/well in 12-well culture plates and differentiated into mature OCs for 7 days. CM was collected from mature OCs. **a,b** BMMs isolated from WT mice were seeded at a density of 1 × 10^4^ cells/well in 96-well culture plates and cultured in α-MEM with OB-CM and 30 ng/ml M-CSF for 7 days. Cells were then subjected to TRAP staining. Negative control (N.C) cells were cultured in α-MEM with 30 ng/ml M-CSF for 7 days and positive control (P.C) cells were cultured in α-MEM with 30 ng/ml M-CSF and 50 ng/ml RANKL for 7 days. **c-d** ADSCs isolated from WT mice were seeded at a density of 1 × 10^4^ cells/well in 96-well culture plates or 2 × 10^5^ cells/well in 12-well culture plates, and exposed to OC-CM for 7 days. **c** Cells were then subjected to ALP staining. **d** The mRNA level was determined using qRT-PCR analysis. **e** The proliferation of ADSCs (3 × 10^3^ cells/well) incubated with OC-CM was measured using the MTT assay. Cells were treated with water-soluble tetrazolium salt solution and incubated at 37˚C for 30 min, and the absorbance was measured at 450 nm. Error bars: mean ± SEM. **p* < 0.05, ***p* < 0.01 (unpaired, two-tailed student’s *t*-test).


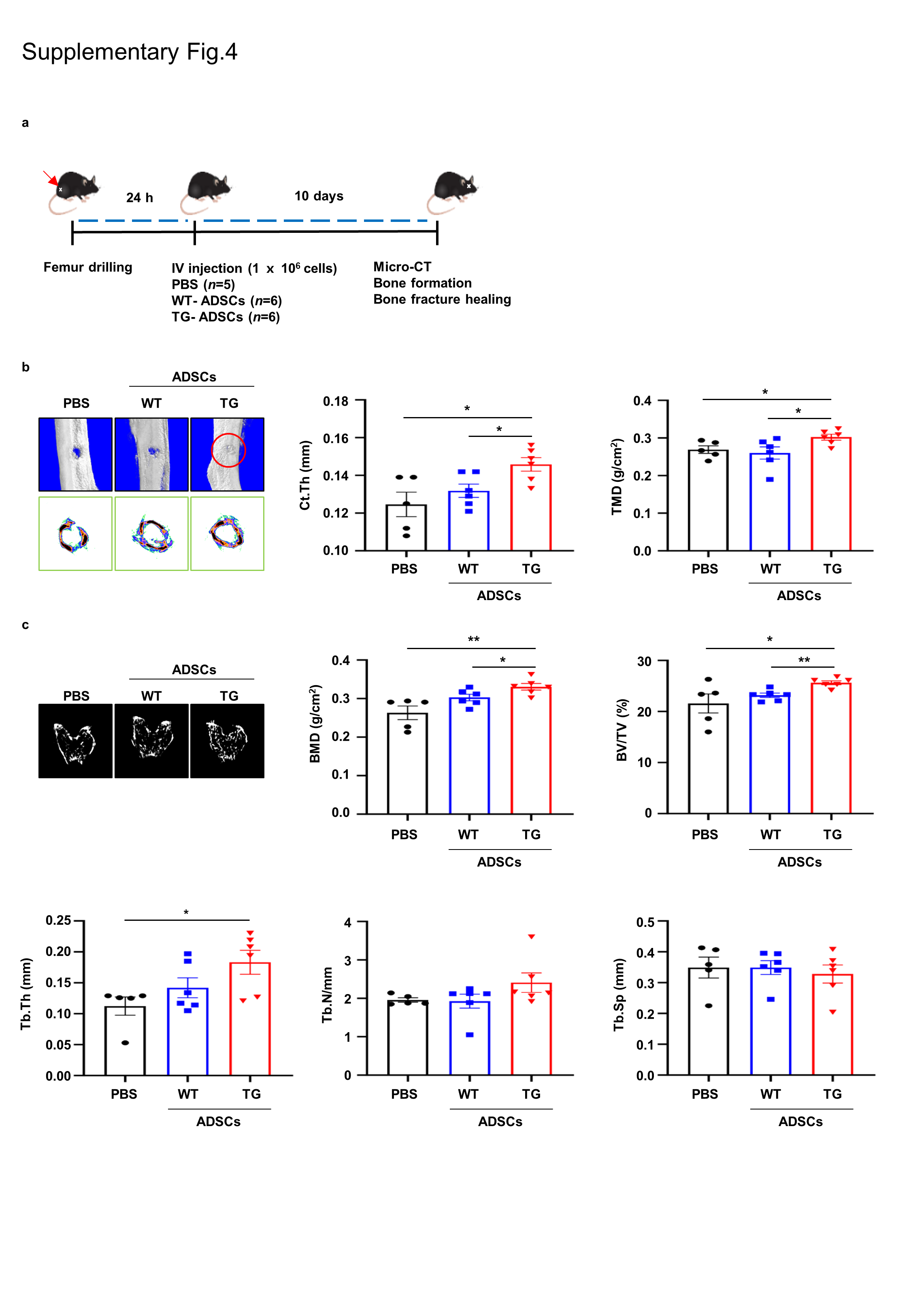


**Supplementary Fig. 4. sLZIP-overexpressing ADSCs induce bone formation and bone healing. a** OVX surgery was performed using WT mice, and each type of ADSC cells (1 × 10^6^ cells/mouse) was injected intravenously after 7 weeks. After 10 days, the mice femur was analyzed μCT. **b** Representative 2D and 3D images were generated from μCT analysis. Quantification of cortical thickness (Ct.Th) and tissue mineral density (TMD) were analyzed using the μCT program (CTAn). **c** Representative 2D images were generated from μCT analysis. Trabecular bone volume per total bone volume (BV/TV), trabecular bone thickness (Tb.Th), trabecular number (Tb. N), and trabecular separation (Tb.Sp) were quantified using the μCT program (CTAn). Error bars: mean ± SEM. **p* < 0.05, ***p* < 0.01 (unpaired, two-tailed student’s *t*-test).

| mRNA | Primer sequence (Forward) | Primer sequence (Reverse) |
| --- | --- | --- |
| *mRUNX2* | AGGGACTATGGCGTCAAACA | GGCTCACGTCGCTCATCTT |
| *mALP* | GGACAGGACACACACACACA | CAAACAGGAGAGCCACTTCA |
| *mCol1a1* | GCTCCTCTTAGGGGCCACT | ATTGGGGACCCTTAGGCCAT |
| *mOsterix* | GAAAGGGAAGCAGAACCATACTATTTG | TCAGTGGTGTGCCTTCATATTCA |
| *mSHPK1* | TGGGCTGTCCTTCAACCTCATACA | AACAGCAGTGTGCAGTTGATGAGC |
| *mSPHK2* | AAGCAAGAGAAAGCTGGTCATC | AGTGACAATGCCTTCCCACTCACT |
| *mSEMA4D* | CCTGGTGGTAGTGTTGAGAAC | GCAAGGCCGAGTAGTTAAAGAT |
| *mEphrinB2* | TCTGTGTCATCGGTTGGCTACGTT | ACAGACGCACAGGACACTTCTCAA |
| *mTRAP* | CTGTGCGACATCAACGAAAGG | CCTTGGGAGGCTGGTCTTAAA |
| *msLZIP* | GAAGCTCTTGGAGAAGGA | TTCAAGACCCTGCTCTCC |
| *sLZIP* | AGCAGCAGCATGTACTCCTCT | AGGCAGCTCCAGCTGGTAAG |
| *mβ-Actin* | AACCCTAAGGCCAACCGTGAAAA | AGGATGGCGTGAGGGAGAGCATA |

**Supplementary Table. 1. Primer sequences used for qRT-PCR.**
